# Editorial

**Published:** 1844-03-23

**Authors:** 


THE MEDICAL EXAMINER.

PHILADELPHIA, MARCH 23, 1844.

### MEDICAL INSTRUCTION IN PHILADELPHIA DURING THE SUMMER MONTHS.

So numerous are the Medical Students who resort to Philadelphia for instruction, and so ample the means for prosecuting their studies advantageously, that a large number of our younger brethren find employment in teaching during the recess of the winter courses. These, far from merely iterating the lectures delivered in the Colleges, often embrace other topics, especially those who are connected with the public charities, and hence contribute in no inconsiderable degree to the enlargement and perfection of our system of medical education. To such an extent indeed are the facilities for gaining information on the various branches of medicine increased in Philadelphia, that it is impossible for one who has not had the opportunity of personal observation within a few years past to form a just conception of the present state of things. The frequent intercourse between this city and London and Paris,—personal, by letters, and the regular exchange of books and journals,—places us almost in juxtaposition with them in regard to all improvements in science and the methods of instruction, and accordingly we find every year a closer approximation in these respects, and perhaps in some instances even a too ready adoption of the novelties in opinion and practice so constantly springing up in these hot-beds of science.

To afford our distant readers some idea of the number of respectable physicians engaged in teaching in Philadelphia during the summer months, we shall note the associations now prominently announced, besides which, however, a number, we cannot say how many, of individuals, devote more or less time in the same way, without connexion with others, but all concurring to maintain for this city the proud pre-eminence which it has always enjoyed as the great seat of medical science in the western world.

#### CLINICAL INSTRUCTION,—PENNSYLVANIA HOSPITAL.

In this noble and time honoured Institution, instruction is given, didactic and clinical, during the summer months, by the physicians, Drs. Stewardson and Pepper, and Drs. Norris and Peace, Surgeons of the Hospital. The cases of recent accidents, such as contusions, fractures, dislocations, &c., are very numerous, in consequence of being received into the wards, when brought from any part of the city and surrounding country, without security or responsibility of payment on the part of those who bring them :—from this source, and the large number of other patients constantly in the house, abundant materials are afforded for complete courses of clinical instruction.

#### PHILADELPHIA DISPENSARY.

The sphere of this Charity extends to the limits of the city proper. Patients who are able to walk, attend at the Dispensary in South Fifth street, every afternoon, and are prescribed for in presence of students of medicine who are desirous of availing themselves of the instruction of the medical officers ; and those who are unable to leave their homes are visited by the physicians of the respective districts in which they reside, who are accompanied at each visit by a suitable number of their pupils. By this arrangement, the students have an opportunity of witnessing the compounding of medicines and performance of various minor operations of surgery every afternoon at the dispensary, and accompanying the visiting physicians to the bedside of patients in the forenoon.

The physicians are Drs. Reese, Tucker, Bryan, Page, Lewis and Stocker,—gentlemen well qualified for the stations they fill.

The obstetric department is under the supervision of Dr. Warrington, well known as an assiduous teacher and practitioner in that branch of our profession. The cases of labour are attended by the advanced students, who have the aid of Dr. W. or his assistant whenever they experience any difficulty or embarrassment.

Drs. Benedict, Reese and Neill, are associated also for giving "practical and clinical instruction and examinations," during the spring and summer months.

Their students have the opportunity of attending patients residing in two districts of the Guardians of the Poor, besides attending the clinics at the Philadelphia Dispensary in the afternoon. They also have cases of labor assigned to them, under the supervision of Dr. Benedict; and have the privilege of attending a course of Lectures by Dr. Reese on Diseases of the Skin, and a course on Diseases of the Eye by Dr. Neill.

Drs. Dunott and Waddrop, give lessons in Practical Anatomy, Surgery, and Surgical Anatomy, at their rooms, in College Avenue.

The physicians and surgeons of the Philadelphia Hospital (Blockley,) and of the Will's Hospital, visit these Institutions regularly on appropriate days, to prescribe for patients and give Instruction to Students who attend for that purpose. The former is one of the largest Hospitals in the World, and consequently offers an extensive field for clinical study : the latter is devoted principally to Diseases of the Eye, and some cases of lameness. As an Eye Infirmary, it is probably the largest and best conducted in this country.

What arrangements exist for Clinical Instruction in the Northern and Southern Dispensaries, we are not advised, nor whether the Clinic lately opened at the University of Pennsylvania, will be continued during the summer season. At the Jefferson Medical College a Clinic is usually held by the Professors, throughout the year, to which *the pupils of the Institution* are admitted without charge.

In addition to all these we have two Associations for giving instruction of a more didactic character: viz. "The Medical Institute," founded by Professor Chapman twenty years since, and lately re-organized, and the "Philadelphia Medical Association," established last year. The advertisement of the former is on the cover of the Examiner, to which reference may be had for further information as to the Lecturers and the subjects taught, terms, &c. &c. The "Philadelphia Medical Association" consists of Drs. Bridges, Wallace, Smith, Meigs, Allen and West. Their course embraces the usual branches taught in Colleges, except the Practice of Medicine, which is not provided for.

### PENNSYLVANIA COLLEGE.

We understand that Dr. David Gilbert, of Gettysburg, has been appointed Professor of Surgery, and Dr. Washington L. Atlee of Lancaster, Pa., Professor of Chemistry. The faculty is therefore now constituted of six professors, three of whom, we believe, are graduates of Jefferson Medical College. At the annual commencement held on the 4th instant, the degree of Doctor of Medicine was conferred on seven gentlemen, pupils of the Institution. We have received the "Valedictory Address delivered before the Graduates," by Professor Henry S. Patterson. It is a sensible and well written production, appropriate to the occasion.

### JEFFERSON MEDICAL COLLEGE.

On another page will be found an account of the annual commencement of this Institution, held on the 20th inst. The Rev. Ashbel Green, D. D., L. L. D., President of the Trustees, conferred the Degree of Doctor of Medicine on one hundred and seventeen gentlemen. Last year the whole number of graduates was forty-seven, which is an increase of seventy, or more than twice that of the former year.

